# Different Associations of Socioeconomic Status on Protein Intake in the Korean Elderly Population: A Cross-Sectional Analysis of the Korea National Health and Nutrition Examination Survey

**DOI:** 10.3390/nu12010010

**Published:** 2019-12-19

**Authors:** Du Ho Kwon, Hyun Ah Park, Young Gyu Cho, Kyoung Woo Kim, Na Hee Kim

**Affiliations:** Department of Family Medicine, Seoul Paik Hospital, Inje University College of Medicine, Seoul 100032, Korea; kwondh1105@gmail.com (D.H.K.); jacobel@hanmail.net (Y.G.C.); kwkimfm@gmail.com (K.W.K.); heee199@naver.com (N.H.K.)

**Keywords:** socioeconomic status, income, education, elderly, animal protein, plant protein

## Abstract

Socioeconomic status affects food choices. This study examined the relationships between socioeconomic status (SES) and animal and plant protein intake in the Korean elderly population whose protein intake is insufficient. We used cross-sectional data from 3512 Koreans aged 60 years or older, who had participated in the Nutrition Survey of the 2013–14 Korea National Health and Nutrition Examination Survey (KNHANES). One day 24-h recall data was used to estimate the daily total, animal, and plant protein intake. Household income and educational attainment were assessed by trained interviewers. After making adjustment, household income was positively associated with animal protein intake with a statistical significance in females (*p* = 0.030) and with a marginal significance in males (*p* = 0.069). However, plant protein intake did not show any significant association. In both sexes, educational attainment was positively associated with animal protein intake (*p* = 0.007 for males, *p* = 0.001 for females). Association of educational attainment with plant protein intake was negative in males (*p* = 0.037) and non-significant in females. (*p* = 0.945). High SES was associated with higher total protein intake and animal protein intake in the Korean elderly. Health policies and nutrition education are needed to improve protein intake of the vulnerable Korean elderly with low SES.

## 1. Introduction

The ageing process increases the risk of frailty [1]. Frail elderly are vulnerable, are prone to dependency, and have reduced life expectancy [2]. Age-related endocrine and metabolic alterations [3] induce body composition changes including progressive loss of muscles and bone mass and acquisition of fat mass [4]. However, protein intake of the elderly has shown beneficial effects on frailty prevention both in cross-sectional [5] and longitudinal studies, [6] mainly through its actions on muscle mass and strength. Protein intake for elderly adults preserves muscle mass, prevents loss of physical functions, and consequently prolongs independent life [7,8].

The RDA (Recommended Daily Allowance) of Korean elderly adults is 0.91 g/kg/day, which is the same as that of young and middle-age adults [9]. However, many studies recommend higher protein intake of at least 1.0–1.2 g/kg/day for the elderly [10,11,12]. A recent Korean study showed that over half of adults over 60 years of age consumed dietary protein that was less than the RDA [13]. Furthermore, more than two thirds of protein consumed was from plant sources [13]. These results suggested inadequate protein intake of the Korean elderly in both quantity and quality.

Dietary intake is affected by socioeconomic status(SES) such as income, education level, and occupational type [14]. However until now, the association between SES and protein consumption has been controversial [14]. For example, studies from Australia, the Netherlands, and low-income and middle-income countries showed positive associations between SES and protein intake, [15,16,17] while an Irish study showed negative associations [18]. There were non-significant associations in Swiss and Japanese studies [19,20]. The inconsistency of association between protein intake and SES may be attributed to the difference in cultural and economic environments, ethnicities, or the type of SES indicators used in the studies [14].

Despite the westernization of the Korean diet, the staple food for Koreans is still rice. Factors affecting protein intake in the Korean elderly are thought to be different from those of western and neighboring Asian countries. However, there are not enough studies to prove a theory. The aim of this study is to investigate the association between SES in terms of household income and educational attainment, and protein intake according to the protein source i.e., plant or animal protein, from a nationally representative sample of the Korean elderly population.

## 2. Materials and Methods

### 2.1. Study Participants

This study is based on the 6th Korea National Health and Nutrition Examination Survey (KNHANES) conducted in 2013–2014. The KNHANES uses two-stage stratified probability sampling to be representative of all the Koreans. The KNHANES is a cross-sectional survey and consists of a health interview survey (household survey, comorbidity, education and economic activities, and health behavior survey), health examination (anthropometric measurements, blood pressure, blood and urine tests) and a nutrition survey (24-h dietary recall, dietary behavior, food stability, dietary supplement, food frequency questionnaire, and nutrition knowledge) [21].

Study subjects included a total of 3512 Koreans aged 60 years or older, of which 1484 were males and 2028 were females. We excluded subjects who reported to consume less than 500 kcal or more than 5000 kcal a day or had any missing data on household income and educational attainment. The non-response rate of household income or education attainment was 0.85%. The study protocol was approved by the Institutional Review Board of Seoul Paik Hospital (IRB No. 2019-02-009).

### 2.2. Household Income and Education Attainment

Well-trained interviewers did the health interview survey. The household income was calculated by dividing the household monthly income by the square root of the household size and then categorizing into quartiles; lower (<591.6$), middle lower (591.6–1388.9$), middle upper (1388.9–2345.5$), and upper (>2345.5$). We divided educational attainment into three categories; ≤9 years (middle school graduate or less), 10–12 years (high school undergraduate or graduate) and 13≤ years (university undergraduate or graduate).

### 2.3. Measurement of Protein Intake

One day 24-h dietary recall was used to estimate the usual daily protein and other nutrients intake. Each food consumed was classified into 19 food groups by the KNHANES standard. Of the 19 food groups, plant protein was taken from grains, potatoes, sugars, beans, legumes, nuts, plants, mushrooms, fruits, seaweeds, drinks and alcohol, condiments, and others (plant), while animal protein was taken from meat, eggs, fish and shellfish, dairy foods, oil (animal), and others (animal). Protein intake was calculated as protein intake in grams per day (g/day) and grams per kilogram body weight per day (g/kg/day). Protein intake adequacy was defined as more than 0.91 g/kg/day, recommended by the 2015 Dietary Reference Intake (DRI) for Koreans [9].

### 2.4. Covariates

Covariates were selected by previous studies [15,22,23]. Weight and height were measured to the nearest 0.1 kg and 0.1 cm. Body mass index (BMI) was calculated as weight (kg) divided by the square of height (m). Smoking status was categorized as either a current smoker or a non-smoker. Alcohol consumption was categorized into three categories; 0, 1, and 2≤ per week, based on how often participants consumed any type of alcohol. We collected data on chronic diseases by self-reporting, including hypertension, dyslipidemia, stroke, ischemic heart disease, osteoarthritis, rheumatoid arthritis, asthma, diabetes, thyroid disease, chronic kidney disease, chronic viral hepatitis, liver cirrhosis, and any types of cancer. Locality of dwelling was divided into two categories; rural and urban. Familial type was divided in 3 categories; living with spouse, living with others, and living alone.

### 2.5. Statistical Analysis

Statistical analysis was performed using SPSS 20 statistical package (SPSS Inc., Chicago, IL, USA) incorporating sampling weight considering the multistage probability sampling design of the KNHANES and the non-responses. All estimates were weighted, so that the results were representative of the Korean population.

Descriptive analyses were performed to examine baseline characteristics for study participants as sample-weighted percentage or mean with standard error (SE). We presented mean and SE of total energy intake, total protein intake, animal protein intake, and plant protein intake according to household income quartile and educational attainment.

General linear modeling was used to evaluate the relationship between SES and total protein, animal protein, and plant protein intake. Analysis was adjusted for age (year), household income quartiles (lower, middle lower, middle upper, or upper), education attainment (≤9 years, 10–12 years, or 13≤ years), current smoking status (yes or no), alcohol intake frequency per week (0, 1, 2≤), presence of chronic disease (yes or no), locality of dwelling (rural or urban), familial type (living with spouse, living with others, or living alone), and total energy intake (kcal). The interaction term between household income and educational attainment was excluded because of their non-significance. Household income quartile and educational attainment were included in the models simultaneously to assess independently. We also estimated odds ratios (ORs, 95% confidence intervals, CIs) of protein intake adequacy by household income quartiles and educational attainment using multivariate logistic regression models while adjusting for age and total energy intake in model 1 and further adjusting for smoking, alcohol, chronic diseases, locality of dwelling and familial type in model 2.

All analyses were stratified by sex. A two-sided *p*-value <0.05 was considered to indicate statistically significance.

## 3. Results

### 3.1. Study Population

A total of 3512 subjects were included in the analysis. Male participants were 43.6 (0.8%). The mean age of subjects was 70.0 (0.2) years in males and 70.3 (0.2) years in females. Roughly two thirds of our participants were from lower or middle lower household income quartiles. The proportions of participants with less than 9 years of education attainment were 59.5 (1.6%) in male and 83.5 (1.2%) in female (Table 1).

### 3.2. Characteristics of Study Population According to Protein Intake

In Table 2, we present protein intake by household income quartiles and educational attainment in both sexes. Males took 13.1 (0.1%) of total energy intake from protein. The mean total protein intake was 1.03 (0.02) g/kg/day while animal protein intake was 0.40 (0.01) g/kg/day. In females, the mean total protein intake was 0.90 (0.01) g/kg/day while animal protein intake was 0.29 (0.01) g/kg/day.

In both sexes, as the household income quartiles and educational attainment increased, the percentage energy from protein (%), total protein intake (g/kg/day), and animal protein intake (g/kg/day) increased. For plant protein intake, females showed a significant positive association with both household income quartile and educational attainment. However, males showed non- significant associations. The increase of protein intake from the lowest to the highest group was greater in animal protein than in plant protein.

### 3.3. Total Protein Intake, Animal Protein Intake, and Plant Protein Intake by SES

**Figure 1:**
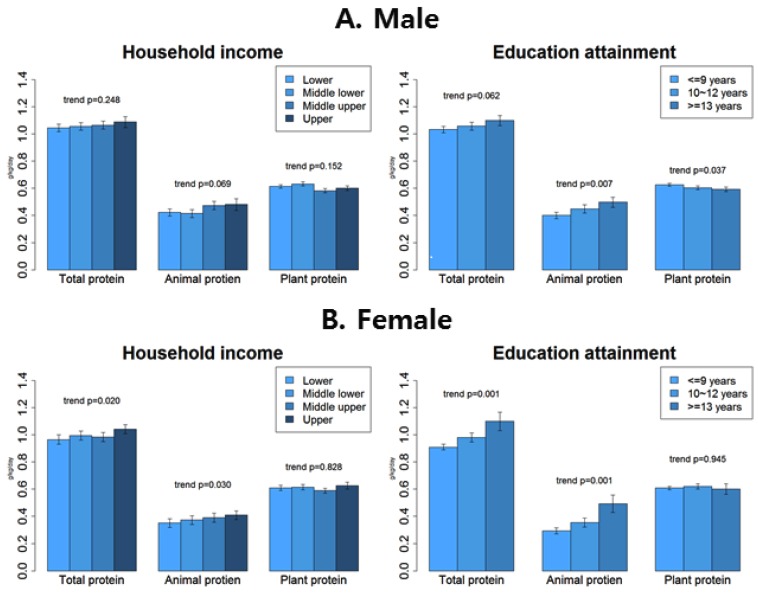
(**A**) Male; (**B**) Female. General linear modeling adjusted for age (continuous), current smoking status (yes or no), alcohol intake frequency per week (0, 1, 2≤), presence of chronic disease (yes or no), locality of dwelling (rural and urban), familial type (living with spouse, living with others, and living alone), and total energy intake (kcal). Household income (lower, middle lower, middle upper, and upper) and education attainment (≤9, 10–12 and 13≤) were adjusted each other.

Figure 1 shows the association between household income quartiles, educational attainment, and protein intake, while controlling age, smoking, alcohol, chronic diseases, locality of dwelling, familial type, and total energy intake.

Household income quartile was positively associated with animal protein intake with a statistical significance in females (*p* = 0.030) and with a marginal significance in males (*p* = 0.069). However, plant protein intake did not show any significant association.

In both sexes, educational attainment was positively associated with animal protein intake (*p* = 0.007 for males, *p* = 0.001 for females). Association of educational attainment with plant protein intake was non-significant in females (*p* = 0.945) and negative in males (*p* = 0.037). The actual decrease of plant protein intake in males from the highest education attainment group to the lowest was minimal (0.03 g/kg/day).

### 3.4. Protein Intake Adequacy by SES

In males, the proportion of protein intake adequacy based on the Korean RDA was 45.8 (2.4%) in the lower household income quartile while it was 59.1 (4.1%) in the upper quartile group. The association between household income quartiles and protein intake adequacy was non-significant (*p* = 0.951). However, educational attainment was significantly associated with protein intake adequacy both in model 1 (*p* = 0.044) and model 2 (*p* = 0.042). The OR of protein intake adequacy in the 13≤ years group was 1.74 (95% CI, 0.99–3.06) compared to the ≤9 years group in model 2.

For females, the proportion of protein intake adequacy was 32.5 (1.7%) in the lower quartile group of household income, while it was twofold in the upper quartile group 61.4 (3.7%). Protein intake adequacy was significantly associated with household income quartile (*p* = 0.002) and educational attainment (*p* = 0.001). The OR of protein intake adequacy in upper quartile household income group was 2.50 (95% CI, 1.50–4.17) compared to the lower quartile group in model 2. Also, the OR of educational attainment of 13≤ years group was 3.21 (95% CI, 1.67–6.17) compared to ≤9 years group in model 2 (Table 3).

## 4. Discussion

Food choice is affected by SES [14,24]. Higher SES groups have been consistently reported to consume more whole grains, fresh vegetables, and fruits, while lower SES groups have been reported to consume more refined sugars, added sugars, fats, and oils. However, reports on the association between SES and protein intake has still been inconsistent.

In a Dutch study, higher educational attainment was associated with higher protein intake [16]. On the other hand, in a French and Irish study, education attainment was negatively associated with protein intake [18,23]. Household income was positively associated with protein intake in Australian adults [15]. However, in an Irish study, household income was negatively associated with protein intake [18]. In Japan, there was non-significant association between household income and protein intake [19]. Meanwhile, a systematic review of low and middle income countries showed that protein intake increased as SES increased [17].

The inconsistency of the relationship between SES and protein intake may be attributed to different socio-cultural environments, characteristics of study participants, and indicators of SES used in the study [19]. In order to resolve this inconsistency, we made two discriminations from previous studies. First, total protein intake was divided into animal and plant protein because protein is consumed through various food sources, but previous studies have not made this distinction. Second, study subjects in most previous studies were young or middle-age adults with sufficient protein intake [15,18,19,23]. However, in this study, we investigated elderly over 60 years of age who were vulnerable to protein deficiency.

### 4.1. Differential Influences of SES Status on Animal and Plant Protein

In this study, indicators of SES were differently associated with animal protein intake and plant protein intake. As SES increased, animal protein intake increased, while plant protein intake did not show significant association except for an unexpected slight negative association with plant protein in males. For Koreans, rice is a staple food which is consumed 15 times per week [25]. Therefore, plant protein intake, which mainly comes from rice, did not vary according to SES. On the contrary, animal protein, which comes from more expensive side dishes including meat, fish, and dairy foods, [26] increased as SES increased. We carefully suggest that the intake of food with low accessibility is greatly affected by the SES of the subject, compared with food with higher accessibility. Also, an increase in animal protein intake from the lowest to the highest SES group was more pronounced in women who consumed less animal protein than men.

The association pattern between total protein intake and SES in Korean adults over 60 years of age was more like that of animal protein intake than that of plant protein intake. This is probably because total protein intake is more affected by animal protein intake which comes from side dishes than plant protein intake which comes from staple foods, especially rice.

Interestingly, educational attainment was negatively associated with plant protein intake in Korean elderly males, though the actual difference of intake between the highest and the lowest group was small (0.03 g/kg/day). For plant protein intake, the most important food group of Korean elderly population was cereals (54.8%), followed by vegetables (14.1%), and legumes (13.4%) without remarkable gender difference [27]. Currently, we do not know the exact explanation of this unexpected negative association because besides refined cereals, plant protein sources like unrefined cereals, vegetables and legumes are commonly consumed healthy food choices for high SES groups. [14] Further studies are needed to evaluate the genuine relationship between educational attainment and plant protein intake.

SES has different effects on animal protein and plant protein intake. Our results suggest that future studies on the relationship between nutrient intake and SES need to consider the food source of the nutrients.

### 4.2. Mechanisms of SES Affect Protein Intake

Although Korea has the twelth largest economy in the world, approximately two thirds of our subjects over 60 years of age had below average household incomes and less than nine years of educational attainment. Similar to low and middle income countries, [17] our study showed that protein intake, particularly animal protein intake was positively associated with household income. This is supposedly because as high household income groups have greater accessibility to expensive foods, they can choose foods with high nutrient density without price barriers [24]. Also, higher education attainment was associated with higher total protein and animal protein intake. Groups with a higher educational level had better nutritional knowledge, which enables them to make healthier food choices [28] and to comply with dietary recommendations [15,29].

There have not been many studies about which SES indicator is more important for food choices. Hassen et al. [23] reported that in under-educated groups, groups with higher household income consumed nutrient-dense foods more, while higher-educated groups consumed nutrient-dense foods regardless of household income. Higher educated people are more likely to accept nutritional knowledge, which is an important factor in overcoming price barriers and making healthy food choices. However, stratified analysis by SES was not performed in our study because of non- significant interaction terms between household income and educational attainment and a decrease in statistical power after stratification.

### 4.3. Recommended Protein Intake for Korean Elderly People

The Korean RDA of 0.91 g/kg/day used in our study for protein adequacy cut-off is very conservative, [6] because RDA is usually based on the nitrogen balance test without considering physiological and biochemical changes with aging. Many societies like the European Society for Clinical Nutrition and Metabolism (ESPEN) and the European Union Geriatric Medicine Society (EUGM) recommend protein intake of more than 1.0–1.2 g/kg/day for elderly adults [10,11]. Even based on current Korean RDA, more than half of Korean adults over 60 years consumed less than the recommended protein intake [13]. If we apply the newly revised protein intake recommendation of more than 1.2 g/kg/day from the Korean Society of Geriatrics in 2018, [12] the proportion of protein intake adequacy drops to 28.7% in men and 20.1% in women. This suggests that deficiency of protein intake is a serious health problem for Koreans aged 60 years or older.

Also, we should consider the protein food source to replenish the protein deficiency. Western studies have so far emphasized plant protein intake, which is related with reducing obesity and CVD mortality, rather than animal protein intake [30,31]. However, we should be cautious in applying study results from countries with excess protein intake to Koreans over 60 years old who are lacking adequate protein intake. For example, Song et al. [31] reported the positive association between animal protein intake and cardiovascular death in the Nurses’ Health Study and the Health Professionals Follow Up study. The mean daily protein intake of the lowest group was 59 g, the middle one was 77 g, and the highest one was 104 g. Animal protein accounted for 77% of the total protein intake. However, in our study, the mean daily protein intake was 56 g, which was lower than the mean of the lowest group of Song et al. [31]. In our study, animal protein intake accounts for only 35% of total protein intake. Therefore, we carefully suggest that animal protein intake such as lean meat, poultry, fish, and low-fat dairy products should be encouraged in Koreans over 60 years of age.

### 4.4. Limitation and Strength

This study has several limitations. First, at least 4–5 days of dietary recall is necessary to assess the usual macronutrients intake of participants [32]. However, the KNHANES used a single one-day 24-h recall because of the application practicability for a large population based survey. A single one- day recall data might be too short a period to evaluate the usual intake of the study participants. Second, self-reported dietary data and SES may have introduced bias. Particularly, SES is one of the most non-responded categories in the health interview [33]. Fortunately, the non-response rates for household income and educational attainment were less than 1% in our population. Protein intake from dietary recall can be validated using biomarkers such as urinary nitrogen excretion test [32]. As far as we understand, there has been no validation study on protein intake of KNHAES. Previous study reported that the expected correlations between daily protein intake and urinary nitrogen excretion are in the region of 0.5 when a single day’s data are used. It becomes 0.95 when 18 days of dietary observation are available. [34] Further studies are needed to evaluate the validity of the KNHANES recall data using biomarkers of dietary intake. Third, there were possibilities of residual confounding because the KNHANES is a cross-sectional survey and our study is a secondary data analysis based on the KNHANES. Fourth, animal and plant proteins could have been further subdivided into sub-categories based on the source of the food. If this distinction had been made, the association between SES and protein intake would have been clearer.

Despite the limitations, to our knowledge this study is the first to present the association between SES and protein intake of Korean elderly people while distinguishing animal and plant protein.

## 5. Conclusions

As the life expectancy of Koreans increases, protein nutrition in elderly people becomes more important to prevent muscle loss and frailty. This study presents that SES assessed by household income and education attainment was positively associated with total protein intake and animal protein intake in elderly population, whose protein intake was far from sufficient. This study is meaningful in that we were able to further identify vulnerable groups with protein deficiency in the Korean elderly. What we now need are health policy researches dealing with nutrition disparities across different SES groups to attenuate the following health disparities. Food price intervention for healthier foods such as food subsidy and nutrition education intervention for healthier foods with low cost might be effective to improve protein intake in Korean elderly population in low SES [35].

## Figures and Tables

**Table 1:** General characteristics of study populations.

Mean (SE) Or Proportion (SE) ^1^	Male	Female	Total
	43.6 (0.8)	56.4 (0.8)	
Age (year)	70.0 (0.2)	70.3 (0.2)	70.1 (0.2)
BMI (kg/m^2^)	23.4 (0.1)	24.4 (0.1)	24.0 (0.1)
Having chronic diseases ^2^ (vs. none)	62.3 (1.5)	75.5 (1.2)	69.7 (1.0)
Alcohol consumption frequency per week			
0	30.8 (1.3)	60.6 (1.3)	47.6 (1.0)
1	38.8 (1.5)	34.4 (1.2)	36.3 (1.0)
2≤	30.4 (1.4)	5.0 (0.6)	16.1 (0.8)
Smoker (vs. nonsmoker)	24.0 (1.3)	2.9 (0.4)	12.1 (0.7)
Urban dwelling (vs. rural)	75.9 (2.6)	76.5 (2.5)	76.2 (2.5)
Familial type			
Living alone	7.5 (0.8)	21.9 (1.0)	15.6 (0.8)
Living with spouse	49.2 (1.6)	29.8 (1.1)	38.3 (1.2)
Living with others	43.3 (1.6)	48.3 (1.4)	46.1 (1.3)
Household income quartiles			
Lower	37.5 (1.6)	46.1 (1.6)	42.3 (1.4)
Middle lower	28.3 (1.4)	27.0 (1.3)	27.5 (1.1)
Middle upper	19.3 (1.2)	15.1 (1.0)	17.0 (0.9)
Upper	14.9 (1.2)	11.8 (1.1)	13.2 (1.0)
Educational attainment (year)			
≤9	59.5 (1.6)	83.5 (1.2)	73.0 (1.2)
10–12	24.9 (1.3)	11.7 (0.9)	17.5 (0.8)
13≤	15.6 (1.3)	4.8 (0.6)	9.5 (0.8)

SE (standard error); BMI (body mass index). ^1^ Values are presented as mean or proportion (standard error). ^2^ Chronic diseases include hypertension, dyslipidemia, stroke, ischemic heart disease, osteoarthritis, rheumatoid arthritis, asthma, diabetes, thyroid disease, chronic kidney disease, chronic viral hepatitis, liver cirrhosis, and any types of cancer.

**Table 2:** Protein intake by household income and education attainment.

	Total Energy Intake, kcal/day	Total Protein Intake, (g/day)	%Energy from Protein, (%)	Protein Intake Per Weight, (g/kg/day)	Total, Animal Protein Intake, (g/kg/day)	Total Plant Protein Intake, (g/kg/day)
Male						
Total	1986.0 (21.9)	65.3 (1.0)	13.1 (0.1)	1.03 (0.02)	0.40 (0.01)	0.63 (0.01)
Household income quartiles						
Lower	1858.9 (27.6)	58.7 (1.4)	12.6 (0.2)	0.95 (0.02)	0.34 (0.02)	0.61 (0.01)
Middle lower	2012.4 (39.4)	65.6 (1.6)	13.0 (0.2)	1.05 (0.03)	0.38 (0.02)	0.65 (0.02)
Middle upper	2079.1 (51.5)	70.0 (2.2)	13.4 (0.3)	1.09 (0.03)	0.46 (0.03)	0.61 (0.02)
Upper	2134.7 (52.9)	75.4 (2.8)	14.1 (0.3)	1.14 (0.05)	0.49 (0.04)	0.63 (0.02)
Trend *p*	<0.001	<0.001	<0.001	<0.001	<0.001	0.563
Educational attainment (year)						
≤9	1929.2 (27.5)	61.4 (1.1)	12.7 (0.2)	0.99 (0.02)	0.35 (0.02)	0.63 (0.01)
10–12	2057.2 (39.9)	68.8 (1.8)	13.3 (0.2)	1.07 (0.03)	0.44 (0.02)	0.63 (0.01)
13≤	2089.0 (59.7)	74.9 (2.8)	14.3 (0.3)	1.15 (0.05)	0.51 (0.04)	0.62 (0.02)
Trend *p*	0.003	<0.001	<0.001	<0.001	<0.001	0.896
Female						
Total	1560.9 (17.0)	49.7 (0.7)	12.6 (0.1)	0.90 (0.01)	0.29 (0.01)	0.60 (0.01)
Household income quartiles						
Lower	1472.8 (19.7)	45.1 (0.9)	12.1 (0.1)	0.83 (0.02)	0.25 (0.01)	0.58 (0.01)
Middle lower	1547.0 (30.3)	49.4 (1.1)	12.8 (0.2)	0.89 (0.02)	0.29 (0.01)	0.60 (0.01)
Middle upper	1697.1 (46.9)	56.0 (1.9)	13.1 (0.3)	0.97 (0.03)	0.35 (0.02)	0.62 (0.02)
Upper	1762.0 (49.1)	60.5 (2.0)	13.6 (0.2)	1.09 (0.04)	0.41 (0.03)	0.68 (0.03)
Trend *p*	<0.001	<0.001	<0.001	<0.001	<0.001	<0.001
Educational attainment (year)						
≤9	1527.7 (18.0)	47.5 (0.7)	12.4 (0.1)	0.86 (0.01)	0.26 (0.01)	0.59 (0.01)
10–12	1666.8 (38.5)	57.4 (1.9)	13.7 (0.3)	1.01 (0.04)	0.37 (0.03)	0.64 (0.02)
13≤	1878.2 (77.9)	68.8 (3.8)	14.7 (0.6)	1.26 (0.09)	0.56 (0.06)	0.70 (0.05)
Trend *p*	<0.001	<0.001	<0.001	<0.001	<0.001	0.016

Values are presented as mean or proportion (standard error).

**Table 3:** The proportion and odds ratio with protein intake adequacy over the RDA *.

		Adequacy (%)	Model 1 OR (CI 95%)	Model 2 OR (CI 95%)
Male				
Household income quartiles				
	Lower	45.8 (2.4)	ref	ref
	Middle lower	53.4 (2.7)	1.04 (0.70, 1.55)	0.99 (0.66, 1.49)
	Middle upper	57.1 (3.2)	1.09 (0.70, 1.69)	1.05 (0.67, 1.66)
	Upper	59.1 (4.1)	1.02 (0.59, 1.75)	0.99 (0.56, 1.77)
	Trend *p*		0.863	0.951
Educational attainment (year)				
	≤9	47.9 (2.1)	ref	ref
	10~12	56.8 (3.0)	1.19 (0.83, 1.70)	1.23 (0.85, 1.77)
	13≤	60.7 (4.3)	1.74 (1.00, 3.05)	1.74 (0.99, 3.06)
	Trend *p*		0.044	0.042
Female				
Household income quartiles				
	Lower	32.5 (1.7)	ref	ref
	Middle lower	40.0 (2.3)	1.43 (1.04, 1.97)	1.45 (1.04, 2.02)
	Middle upper	45.3 (3.2)	1.13 (0.74, 1.74)	1.17 (0.75, 1.83)
	Upper	61.4 (3.7)	2.41 (1.49, 3.90)	2.50 (1.50, 4.17)
	Trend *p*		0.002	0.002
Educational attainment (year)				
	≤9	36.7 (1.5)	ref	ref
	10–12	48.8 (4.0)	1.26 (0.80, 1.99)	1.29 (0.80, 2.06)
	13≤	72.5 (4.6)	3.30 (1.70, 6.40)	3.21 (1.67, 6.17)
	Trend *p*		0.001	0.001

OR (odds ratio); RDA * 0.91 g/kg/day, recommended daily allowance. Values are presented proportion (standard error) or odds ratio (95% CI). Model 1 adjusted for age (continuous) and total energy intake (kcal). Model 2 adjusted for age (continuous), smoking (yes or no), alcohol (0, 1, 2≤), chronic diseases (yes or no), locality of dwelling (rural and urban), familial type (living with spouse, living with others, and living alone) and total energy intake (kcal).
